# A taxonomic revision of *Limnobaris* Bedel in the strict sense (Coleoptera, Curculionidae, Baridinae), with particular emphasis on the species found in China

**DOI:** 10.3897/zookeys.416.7164

**Published:** 2014-06-16

**Authors:** Jens Prena, Boris Korotyaev, Zhiliang Wang, Li Ren, Ning Liu, Runzhi Zhang

**Affiliations:** 1 Key Laboratory of Zoological Systematics and Evolution, Institute of Zoology, Chinese Academy of Sciences, Beijing 100101, P. R. China; 2 Zoological Institute, Russian Academy of Sciences, Universitetskaya nab., 1, St. Petersburg 199034, Russia

**Keywords:** Weevil, sedge, distribution, life history, parasitoid, Palaearctic

## Abstract

The genus name *Limnobaris* Bedel is applied in a restricted sense to baridine weevils with a covered pygidium and non-prominent, decussate mandibles which occur on sedges in the Palaearctic Region and immediately adjacent parts of tropical Southeast Asia. *Calyptopygus* Marshall and *Pertorcus* Voss are **syn. n.** of *Limnobaris*. Some species from Africa and the Americas are maintained provisionally in *Limnobaris* in the widest sense but will need to be transferred to other genera in future studies. A total of eleven species is recognized in Asia, two of which are widespread and occur also in the Western Palaearctic Region. *Limnobaris martensi* Korotyaev **sp. n.** is described from Nepal. *Pertorcus tibialis basalis* Voss is raised to species rank, as *L. basalis* (**stat. prom.**). New or reestablished synonyms are *L. dolorosa* (Goeze) (= *L. jucunda* Reitter, = *L. koltzei* Reitter), *L. tibialis* (Voss) (= *Pertorcus tibialis pilifer* Voss) and *L. t-album* (Linnaeus) (= *L. bedeli* Reitter, = *Baridius crocopelmus* Gyllenhal, = *L. sahlbergi* Reitter, = *L. scutellaris* Reitter, = *Baris t-album sculpturata* Faust). *Calandra uniseriata* Dufour is considered a junior synonym of *Sitophilus oryzae* (L.) (**syn. n.**). A key for identification and a distribution map are provided.

## Introduction

Baridinae are hyperdiverse, oligophagous weevils with a worldwide distribution. Many are uniformly oblong-ovate and notoriously poor in taxonomically useful characters. A particular problem is that their higher classification is based largely on poorly known exotic species with aberrant shapes and bizarre character formations, while at the same time leaving the bulk of nondescript species in an unwieldy, phylogenetically meaningless disarray. The situation has escalated over the past century because regionally conducted studies increasingly failed to associate new collections and observations with previously published information. Even the relatively well-studied species of the temperate northern hemisphere are in need of much work (Morimoto and Yoshihara 1996, Anderson 2002).

**Figure 1.**
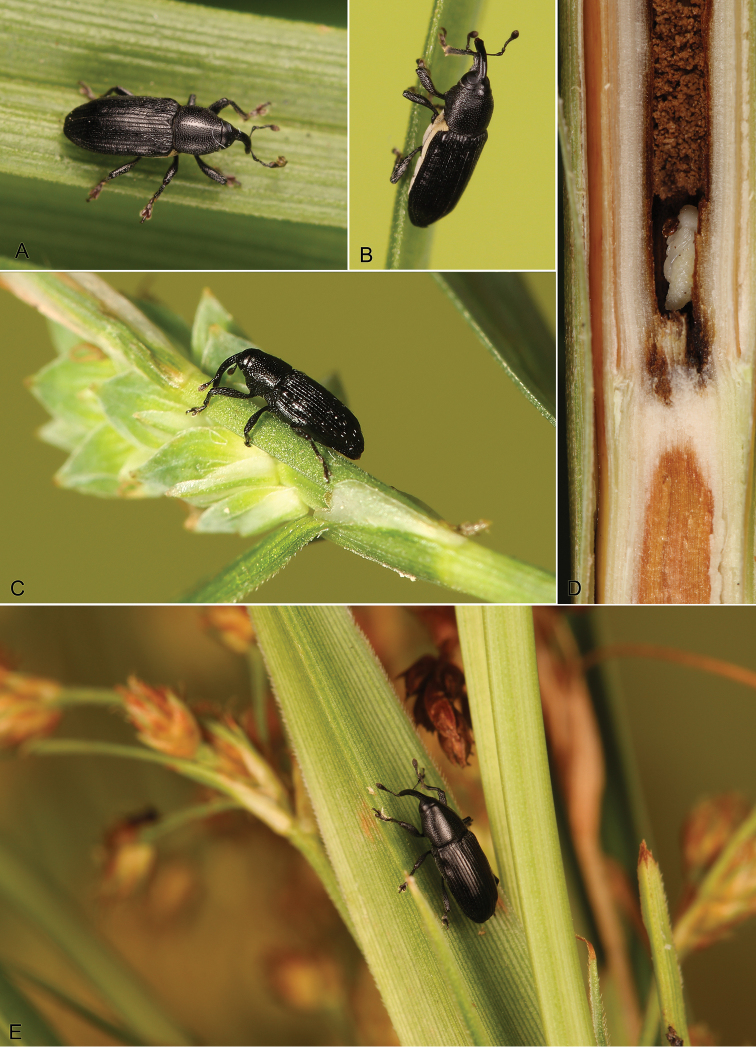
Live habitus images of *Limnobaris* species from China (including type species of *Calyptopygus* and *Pertorcus*). **AB** *Limnobaris dolorosa* (3.6 mm) on *Carex* and *Scirpus* in Changbaishan, Jilin Province **C** *Limnobaris tibialis* (2.8 mm) on *Carex* in Wuyishan, Fujian Province **DE** Pupa and adult (3.7 mm) of *Limnobaris elliptica* in/on *Scirpus wichurai* in Xiaozahe, Yunnan Province. Photos by Wang Zhiliang (**A, B, D, E**) and Ding Liang (**C**).

The present study is concerned with an ill-defined complex of small, slender, usually black species associated with Cyperaceae (Fig. 1). Bedel (1885) was the first to place two Western Palaearctic species in a separate genus named *Limnobaris* Bedel, which differs from other European baridines by having the pygidium completely covered by the elytral apices. This simple but useful character was accepted readily by contemporary entomologists: Reitter (1888) honored Bedel for his keen observation with the patronym *Limnobaris bedeli*; Casey (1892) adopted the concept for Nearctic species, Hartmann (1904) for an African species and Faust (1896), Champion (1908, 1909) and Hustache (1932) for Neotropical species. However, this worldwide concept no longer functioned when Casey (1920, 1922) applied new generic names to North American and Brazilian species in his private collection but ignored many others not immediately available to him. In addition to these still existing but obsolete generic placements, further problems have evolved around certain East Asian species, which currently are placed in three genera. Most share a distinct ventromedian tooth on the male protibia and all lack the dense, lateral vestiture of the type species, *Limnobaris t-album* (Linnaeus). Marshall (1948) described the genus *Calyptopygus* for a species with the above characters from the Myanmar-China border region but did not recognize its similarity to *Limnobaris*. Likewise, Voss (1953) described *Pertorcus* for a species from China’s Fujian Province, erroneously stating it has only six funicular segments. Korotyaev (1982) described *Limnobaris kabakovi* from Vietnam and was the first to make a connection between *Limnobaris* and *Pertorcus*, but was misled by Voss’ faulty description of the antenna. Yoshihara and Morimoto (1994) did not recognize the latent problem so *Calyptopygus* and *Pertorcus* were disregarded in their revision of East Asian *Limnobaris* species. Subsequent regional studies used the name *Calyptopygus* without providing diagnostic criteria (Yoshihara and Morimoto 1997) or their criteria made *Calyptopygus* and *Limnobaris* paraphyletic to each other (Morimoto and Yoshihara 1996, Zherikhin 1997). Kojima and Morimoto (2004) and Prena (2011) merely cataloged the status quo. The primary objective of this study is to resolve the taxonomic conflict in the current usage of the names *Limnobaris*, *Calyptopygus* and *Pertorcus*, and to provide a means for the identification of the species likely to be found in China. As this covers almost all known species of the genus in the restricted sense adopted herein, our results may help future students of the *Limnobaris* complex in other biogeographical regions.

## Material and methods

This study deals with the European and Asian species currently placed in the genera *Limnobaris*, *Calyptopygus* and *Pertorcus*, but ignores one nominal *Limnobaris* species from Africa and 26 from the Americas. We have examined most of them and concluded that the taxonomic problems are too complex to be treated here. Rather than adding to the confusion with incomplete and preliminary new generic placements, we decided to leave them provisionally in *Limnobaris* sensu lato.

Several species treated herein have been documented in great detail by Dieckmann (1991) and Yoshihara and Morimoto (1994, 1997), and we have used their results and observations directly without always checking the types or even representative material. However, we studied the types of most remaining species and refer in the text to their and other specimens’ current repositories with the following acronyms: AKMB, Zoologisches Forschungsmuseum Alexander Koenig, Bonn, Germany; BMNH, The Natural History Museum, London, United Kingdom; BPBM, Bernice P. Bishop Museum, Honolulu, Hawaii; CWOB, Charles W. O’Brien personal collection, Green Valley, Arizona, U.S.A.; ELKU, Entomological Laboratory, Kyushu University, Fukuoka, Japan; HNHM, Hungarian Natural History Museum, Budapest, Hungary; IEGG, Università di Bologna, Istituto di Entomologia “Guido Grandi”, Italy; IZCAS, Institute of Zoology, Chinese Academy of Sciences, Beijing, China; JPPC, Jens Prena personal collection, Berlin, Germany; LSUK, Linnean Society, London, United Kingdom; MCZ, Museum of Comparative Zoology, Harvard University, Cambridge, U.S.A.; MNHN, Muséum National d’Histoire Naturelle, Paris, France; MNKB, Museum für Naturkunde, Berlin, Germany; MZH, Finnish Museum of Natural History, Helsinki, Finland; NHRS, Naturhistoriska riksmuseet, Stockholm, Sweden; OMPB, Osservatorio per le Malattie delle Piante per la Regione Emilia-Romagna, Bologna, Italy; SDEI, Senckenberg Deutsches Entomologisches Institut, Müncheberg, Germany; SFFM, Senckenberg Naturforschendes Museum, Frankfurt am Main, Germany; SMNS, Staatliches Museum für Naturkunde Stuttgart, Germany; SNSD, Senckenberg Naturkundliche Sammlungen Dresden, Germany; ZAFU, Institute of Forest Protection, Zhejiang A & F University, Zhejiang, China; ZIN, Zoological Institute of the Russian Academy of Sciences, St. Petersburg, Russia; ZIUH, Zoologisches Institut der Universität Hamburg, Germany.

Because of our emphasis on Chinese fauna, we list under “Material examined” only specimens collected not further than 1000 km away from the Chinese mainland. Collecting trips were made by us to Fujian, Jilin and Yunnan in 2013 to obtain biological data and fresh specimens for morphological and future molecular comparison of regional populations. For the map in Fig. 13, we combined our records with collecting data published by Zherikhin (1972), Egorov (1976, 1977), Yoshihara and Morimoto (1994), Hong et al. (2000, 2011), Legalov (2009) and in original descriptions. Geographic distributions were explored with GoogleEarth directly from the graphical user interface of JP’s database via GoogleEarth API. The map presented in this paper was generated with PanMap (www.pangaea.de). Illustrations were prepared from images taken with a Micropublisher 5.0 RTV digital CCD camera mounted on a Zeiss SteREO Discovery V12 or a Canon EOS 650D on a Leica DM2500 compound microscope. Aldus Freehand was used for vector graphics and Adobe Photoshop for pixel-based artwork. Total length was measured from the anterior margin of eye to the abdominal apex in dorsal view with an ocular micrometer in a dissecting microscope.

## Results and discussion

### Delineation and classification of *Limnobaris*

The present ambiguities in the usage of the name *Limnobaris* relate to two different issues. The first is associated with its application to species in the New World and tropical Africa, the second with the placement of very similar East Asian species in three different genera.

The taxonomy of the American and African species presently placed in *Limnobaris* is not the objective of this study. Their descriptions were based on Casey (1892), who noted that *Limnobaris* (in the widest sense) differs from most New World baridines with a covered pygidium by non-prominent, decussate mandibles. When Casey (1920) substituted *Limnobaris* in his earlier sense with new genera, species not represented in his own collection remained in *Limnobaris* and only a few of them have been transferred to other genera by Buchanan (1932), Kuschel (1983) and Prena (2013). The remaining American *Limnobaris* species belong to eight poorly defined, primarily Neotropical genera and need revision. *Limnobaris lineigera* Hartmann, from Tanzania, is excluded from this paper for the same reason; it is misplaced in *Limnobaris* and needs to be compared with African material. We maintain the respective New World and African species in *Limnobaris* sensu lato and refer the issue to the next revising authors.

The chief problem in the current usage of the name *Limnobaris* in the Palaearctic Region is the paraphyly to *Calyptopygus* and *Pertorcus*. Voss (1953) stated incorrectly that *Pertorcus tibialis* has only six funicular segments. However, this species is part of a morphologically homogeneous complex which includes also *Limnobaris albosparsa* Reitter and the type species of *Calyptopygus*, *Calyptopygus ellipticus* Marshall. This makes *Pertorcus* at least a junior subjective synonym of *Calyptopygus*, while the distinctness of *Calyptopygus* and *Limnobaris* needs to be addressed. Morimoto and Yoshihara (1996) distinguished between *Limnobaris* and *Calyptopygus* because they observed differences in (1) the length and shape of the rostrum, (2) the length proportions of the funicular segments and (3) how the mesosternum connects to the metasternum. These criteria seem to have been taken from *Calyptopygus kumei* Yoshihara & Morimoto, 1997 rather than from the type species, *Calyptopygus ellipticus*. At least (2) does not hold for *Calyptopygus ellipticus*, (1) only for females and (3) does not seem to work at all. Zherikhin (1997) did not say why he transferred *Limnobaris albosparsa* to *Calyptopygus* but he obviously took into account the male protibial tooth (Figs 3–5) and perhaps vestiture. These two criteria separate *Limnobaris t-album*, *Limnobaris dolorosa* and *Limnobaris japonica* (all with edentate male protibia and dense ventrolateral vestiture) from the remaining species, which have dentate male protibiae (except *Calyptopygus kumei*) and sparse ventrolateral vestiture. All included species are associated with sedges as far as is known (see taxonomic treatment further below for data and references). *Limnobaris* and *Calyptopygus* each have species that develop either in *Carex* or *Scirpus*, or both. Our preliminary molecular data of five species indicate that *Limnobaris t-album* and *Limnobaris dolorosa* nest inside the *Calyptopygus*/*Pertorcus* clade and are derived from ancestors with sparse vestiture and a long rostrum. Until more species are sequenced and our understanding of the relationships between the relevant Old and New World groups has improved, we suggest treating the presently known eleven Eurasian species in a single genus and consider *Calyptopygus* Marshall and *Pertorcus* Voss as subjective junior synonyms of *Limnobaris* Bedel (**syn. n.**).

The unresolved relationships of the *Limnobaris* complex in the wide sense affect approximately 25 genera worldwide, 160 currently valid species-group names and at least two family-group names. Most of these genera occur in the Americas and are placed in Zygobaridina Pierce, 1907, currently with Limnobaridina Casey, 1922 and Torcina Bondar, 1943 included as junior synonyms (Prena 2012). The usage of Zygobaridina derives from obsolete views expressed by Jekel (1865), Lacordaire (1865) and Casey (1892). The name had been ignored in weevil systematics until Alonso-Zarazaga and Lyal (1999) used it to replace Centrinina Jekel, 1865, a junior homonym preoccupied in the sharks. However, Zygobaridina becomes paraphyletic to the more senior Leptoschoinina Lacordaire, 1865 when the exposure of the pygidium is disregarded (Prena 2008). The matter is complicated further by species in *Anacentrinus* Buchanan, 1932 (currently synonymized with *Apinocis* Lea, 1927), which develop in grasses (Barber 1927, Böving 1927, Buchanan 1932, Wille 1934, Bryson 1941) but also in sedges (J. Prena, unpubl. data) and connect the *Limnobaris* complex morphologically and ecologically to Madopterina Lacordaire, 1865, still another family-group name with nomenclatural priority. Many of the American taxa relevant for a phylogenetic reconstruction are fraught with taxonomic problems and bristle with unrecognized synonyms and misinterpreted names. As long as these issues remain unresolved and nominal types for family-group names are either excluded or misidentified in phylogenetic studies, the prevailing confusion will continue to escalate.

### Misplaced Palaearctic species

*Calandra uniseriata* Dufour, 1843 was described from the French Pyrenees. The author characterized briefly the genus and placed the species between *Sphenophorus* Schönherr and *Sitophilus* Schönherr in the modern sense. However, Heyden et al. (1891) transferred *Calandra uniseriata* to *Limnobaris*, where it remained until L. Zerche (in Dieckmann 1991) questioned this placement. On our request, H. Perrin kindly searched for the specimen in the Dufour Collection at MNHN and confirmed its absence therein (*in litt*., 2.viii.2013). Likewise, we found no vouchers in the Heyden material in SDEI and SFFM. Because there is no clear evidence for a misplacement of *Calandra uniseriata* in Dryophthorinae and the type seems to be lost, we tentatively transfer the species to *Sitophilus* and consider it a synonym of *Sitophilus oryzae* (L.) (**new placement, syn. n.**). This placement is made with the purpose to protect the generally accepted name for the cosmopolitan storage pest, *Sitophilus zeamais* (Motschulsky), at least until all relevant senior nominal taxa have been reviewed.

## Synopsis of Palaearctic species

### Limnobaris albosparsa

Taxon classificationAnimaliaColeopteraCurculionidae

Reitter, 1910

Limnobaris albosparsa Reitter, 1910: 203. Holotype, sex not determined, Ussuri, Russia (HNHM).
Calyptopygus albosparsus : Zherikhin 1997: 3.

#### Diagnosis.

Small size (2.7–3.2 mm) and scattered, squamiform, appressed setae on the elytron make *Limnobaris albosparsa* a distinctive species around the Eastern Sea (Sea of Japan). The aedeagus is identical to that of the more southern *Limnobaris tibialis* except for its somewhat wider base (Fig. 10 vs. Yoshihara and Morimoto 1994: 451), and the two species usually have a sharply pointed tooth on the ventral edge of the male protibia (Fig. 5). However, they are allopatric (Fig. 13) and *Limnobaris tibialis* has erect rather than appressed setae on the elytron. Other small species, like *Limnobaris basalis*, *Limnobaris elliptica* and *Limnobaris martensi*, have a blunt male protibial tooth and lack scattered squamiform setae on the elytron.

#### Distribution.

The species has been reported from Japan (Honshu), Russia (Khabarovsk and Primorsky krajs) and South Korea (Reitter 1910, Egorov 1976, Yoshihara and Morimoto 1994, Hong et al. 2000, 2011, Legalov 2009). It may occur also in the Chinese provinces Heilongjiang and Jilin (Fig. 13).

#### Biology.

Nojima (in Yoshihara and Morimoto 1994) found specimens on *Carex dickinsii* Franch. and Savat. in Yoshikawa, Japan. Most known collecting dates fall between the middle of May and the end of June. Three specimens were collected in August and September (ZIN, data below) and indicate that the new generation emerges in the same year and overwinters in the adult stage.

#### Material examined.

RUSSIA. Primorsky Kraj: Golubiny Utes, 27.viii.1988 (ZIN 1); Kamen'-Rybonov 20 km NW, 26.vi.1974 (ZIN 3); Kamenushka, 12.vi.1989 (ZIN 1); Khanka Lake, 20 km W Spassk, 12.vi.1989 (ZIN 1); Provalovo, 12.ix.1982 (ZIN 2).

### Limnobaris babai

Taxon classificationAnimaliaColeopteraCurculionidae

Chûjô & Morimoto, 1959

Limnobaris babai Chûjô & Morimoto, 1959: 153. Holotype female, Kurokawa, Echigo, Honshu, Japan (ELKU).

#### Diagnosis.

The Japanese *Limnobaris babai* is very similar to *Limnobaris elliptica* from Myanmar and South China. These two species can be distinguished from all others by the blunt ventromedian process on the male protibia and almost glabrous elytra. *Limnobaris babai* is on average larger than *Limnobaris elliptica* (3.5–4.5 mm vs. 3.2–3.8 mm), has shorter setae on the profemur, an apically rounded penis and the female protibia has a moderate ventromedian projection which is more subtle in *Limnobaris elliptica*. However, we have not compared specimens of the same size and these differences may not always hold. *Limnobaris basalis*, another morphologically similar species from Fujian, is smaller (2.3–3.1 mm) and has a longer rostrum.

#### Distribution.

The species occurs in Honshu and Kyushu, Japan (Yoshihara and Morimoto 1994).

#### Biology.

Adult weevils have been collected from *Carex* sp. (Yoshihara and Morimoto 1994).

#### Material examined.

JAPAN. Saitama Pref., Urawa City, 23.v.1998 (JPPC 4).

### Limnobaris basalis

Taxon classificationAnimaliaColeopteraCurculionidae

(Voss, 1958) stat. prom. & comb. n.

Pertorcus tibialis basalis Voss, 1958: 76. Holotype female, Kuatun [Guadun], Fujian, China (AKMB).

#### Diagnosis.

*Limnobaris basalis* is a small species (2.3–3.1 mm) with white, squamiform setae on the elytral base. Males can be distinguished from the equally small *Limnobaris albosparsa* and *Limnobaris tibialis* by the shape and position of the ventral process on the protibia (Fig. 3). Abraded females of *Limnobaris basalis* and *Limnobaris tibialis* may be distinguished by body proportion (*Limnobaris basalis* is slightly stouter) and host association, *Limnobaris basalis* and *Limnobaris albosparsa* by allopatry. Other similar but allopatric species are *Limnobaris babai* (Japan) and *Limnobaris elliptica* (South China, Myanmar). They are larger, have smaller and fewer scales on the elytral base and a shorter rostrum.

#### Distribution.

The species is known from several sites in Fujian Province, China (Fig. 13).

#### Biology.

The overwintered weevil appears on *Scirpus* in early April and feeds on the leaves. In late April, when the plant flowers in Fujian, we observed numerous specimens on the inflorescence. The larva develops in the culm, apparently most successfully in the basal, often submerged internode. Occupants of more distal internodes frequently were parasitized by an unidentified species of the *Eupelmus* (s. str.)*urozonus* Dalman group (Eupelmidae, Hymenoptera). Pupation starts in June and newly eclosed adults appear in July. In middle July 2013, we found mostly larvae, approximately one third pupae and one eclosed adult in the culms we dissected.

#### Notes.

*Pertorcus tibialis basalis* is transferred here to *Limnobaris* and raised to full species rank based on plant association, molecular data and morphological details of the protibia, genitalia and vestiture.

#### Material examined.

CHINA. Fujian: 建阳坳头[?]田坵 [?tianqiu, Aotou, Jianyang], 25.iv.1965 (IZCAS 1); 建阳坳头三板桥 [Sanban Bridge, Aotou, Jianyang], 19.iv.1965, 26.iv.1965, 18.v.1991 (IZCAS 3); Daanyuan, Wuyi Mts., 24.–27.iv.2013 (BMNH 4, IZCAS 17, JPPC 11, ZIN 4), 16.–19.vii.2013 [larvae, pupae, 1 adult, parasites] (IZCAS, JPPC); Guadun, Wuyi Mts., 1.iv.1938 (PT), 2.iv.1938 (HT), 7.iv.1938 (4x), 8.iv.1938 (5x), 12.iv.1938 (2 PT), 15.iv.1938, 16.iv.1938, 19.iv.1938, 25.iv.1938 (5x), 27.iv.1938, 5.v.1938, 6.v.1938 (1 + 1 PT), 7.v.1938 (AKMB 25); 大竹栏 [Dazhulan], 4.vii.1965 (IZCAS 1); Guangze, 5.ix.1937 (AKMB 1); 建阳黄坑新历 [Xinli, Huangkeng, Jianyang], 25/27.v.1965 (IZCAS 2); 建阳黄坑 [Huangkeng, Jianyang], 10.–13.iv.1965 (IZCAS 4); 建阳将乐龙栖山 [Longqi Mts., Jiangle, Jianyang], 19.iv.1965, 3.vii.1965, 17.–19.v.1991 (6x), 1.viii.1991 (IZCAS 9); 梅花山双车村 [Shuangche Village, Meihua Mts.], 6.xi.2008 (IZCAS 1); 黄坑大竹篮先峰岭 [Xian Fengling, Huangkeng Dazhulan, Jianyang], 28.v.1960 (IZCAS 2).

### Limnobaris dolorosa

Taxon classificationAnimaliaColeopteraCurculionidae

(Goeze, 1777)

“17. Curculio”. Geoffroy (1762: 285) [nomenclaturally unavailable work, species names not binominal].
Curculio dolorosus Goeze, 1777: 411. Description from Geoffroy (1762) in combination with available name. Neotype designated by Dieckmann (1991: 305), male, Lagny, France (MNHN).
Curculio funereus Geoffroy in Fourcroy 1785: 121. Description from Geoffroy (1762) in combination with species name. Objective synonym of *Curculio dolorosus* Goeze.
Curculio dolorosus Gmelin, 1790: 1804. Description from Geoffroy (1762) in combination with species name. Homonym and objective synonym of *Curculio dolorosus* Goeze.
Curculio funereus Herbst, 1795: 164. Lectoype designated by Dieckmann (1991: 307), male, vicinity of Brunswick [Braunschweig], Germany (MNKB). Homonym of *Curculio funereus* Geoffroy, synonymized with *Curculio dolorosus* Goeze by Dieckmann (1991).
Baris pilistriata Stephens, 1831: 10. Lectoype designated by Dieckmann (1991: 308), sex not determined, vicinity of London, England (BMNH). Synonymized with *Curculio dolorosus* Goeze by Dieckmann (1991).
Limnobaris koltzei Faust, 1892: 333. Syntypes at least 6, Dalmatia (SDEI, SNSD). Synonymized with *Curculio dolorosus* Goeze by Dieckmann (1991).
Limnobaris koltzei Reitter, 1895: 31. Syntypes 2, Dalmatia (SDEI). hom. n., syn. n.
Limnobaris barbiellinii Leoni, 1907: 196. Holotype, sex not determined, Rome, Italy (probably in IEGG or OMPB). Synonymized with *Curculio dolorosus* Goeze by Dieckmann (1991).
Limnobaris jucunda Reitter, 1910: 202. Holotype, sex not determined, Ussuri, Russia (HNHM). syn. n.

#### Diagnosis.

*Limnobaris dolorosa* has characteristic ventrolateral vestiture of dense, squamiform setae, which occurs also in *Limnobaris t-album* and *Limnobaris japonica*. All three species can be distinguished from each other based on details of the male genitalia and vestiture. In addition, *Limnobaris japonica* is allopatric (see under this species for further details). The safest way to separate *Limnobaris dolorosa* from *Limnobaris t-album* is the short and wide penis (Figs 6, 7). Moreover, *Limnobaris dolorosa* has evenly dense vestiture on the entire flank, whereas *Limnobaris t-album* usually has less dense vestiture on the first ventrites and the metasternum.

#### Notes.

Specimens with a dolorosa-type of aedeagus show regional variation in size, body shape, vestiture and surface sculpture. Dieckmann (1991) mentioned disjunct, aberrant populations of unusually large specimens, one of which was found on *Cladium mariscus* (L.) Pohl rather than *Carex*. This scarce material was collected at sites with mild winters and cool summers (Swedish Baltic islands and Adige River valley in South Tyrol). Our own study showed that the East Asian *Limnobaris jucunda* is a population with a dolorosa-type of aedeagus that is morphologically indistinguishable from European *Carex*-associated specimens. However, small, densely squamose specimens with the same male genitalia occur in Transbaikal, Mongolia and adjacent Northeast China. Further studies with inclusion of molecular and ecological data of geographically representative material are needed to better understand the nature of this variation. We therefore follow Dieckmann (1991) and consider *Limnobaris dolorosa* (and, likewise, *Limnobaris t-album*) as a morphologically and ecologically polytypic species.

#### Distribution.

The range of *Limnobaris dolorosa* extends from Western Europe (without Iberian Peninsula) to the Pacific coast (apparently without offshore islands, although Legalov (2010) reported the species from the Kuril Islands, possibly a misidentification of *Limnobaris japonica*). In China, the species has been found in Heilongjiang, Inner Mongolia and Jilin (Fig. 13).

#### Biology.

Because of taxonomic confusion with *Limnobaris t-album*, published life history data are unreliable and need verification. Specimens of the typical size range occur on *Carex rostrata* Stokes ex With. in Scotland (Cawthra 1957; see Morris 2009 for weevil identification) and Northern Germany (J. Prena, unpubl. data). Palm (1957) found very large specimens (up to 7 mm) on *Cladium mariscus* in the Baltic islands Öland and Gotland. Hoffmann (1955) mentioned *Scirpus sylvaticus* L. as a host plant. In Jilin Province, we found *Limnobaris dolorosa* in mixed stands of *Scirpus* and tall *Carex* species (mostly *Carex rhynchophysa* C. A. Meyer, some *Carex drymophila* Turczaninow ex Steudel), occasionally in pure *Carex* stands. Feeding occurred on leaves of *Carex rhynchophysa* and *Scirpus*. Life history data published by Kleine (1910), Hoffmann (1955) and Cawthra (1957) on *Limnobaris t-album* almost certainly apply to *Limnobaris dolorosa*. According to Kleine (1910), the larva develops in the stem of *Cladium mariscus*. Cawthra (1957) reported that eggs are laid in the basal part of the leaf of *Carex rostrata* and that the larva bores down to the rhizome, overwinters, and pupates inside the plant in late May. Hoffmann (1955) described an almost identical development in *Schoenoplectus lacustris* (L.) Palla.

#### Material examined.

CHINA. Heilongjiang: 哈尔滨 [Haerbin], 31.v.1943 (IZCAS 2); 虎林 [Hulin], 9.vi.1971 (IZCAS 3); 虎林虎头 [Hutou, Hulin], 9.vi.1971 (IZCAS 2); 二層甸子 [Ercengdianzi, =Yuquan], 15.vi.1941, 30.vi.1941 (3x) (IZCAS 4). Inner Mongolia: 海拉尔嵯岗 [Cuogang, Hailaer], 22.vi.1994 (IZCAS 1). Jilin: Changbai Mts., Dongwo, 8.–11.vi.2013 (IZCAS 11, JPPC 8). MONGOLIA. Dornod Aymag: Duro-Nur Lake [Dörgön núr], 15 km W Khukh-Nur Lake, 28.vi.1976 (ZIN 1). RUSSIA. Zabajkal’skij Kraj: Darasun, 28.vi.1975 (ZIN 2); Soktuj, 23.vi.1925 (ZIN 1). Irkutskaya Oblast: Irkutsk, vii.1994 (JPPC 1). Kemerovskaya Oblast: Tyazhin, 17.vi.1958 (IZCAS 1). Republic of Buryatia: Kjakhta District, NW Kiram, 27.vi.1999 (JPPC 1); Barun-Torej Lake, Ulza River, 29.vi.1925 (ZIN 2).

### Limnobaris elliptica

Taxon classificationAnimaliaColeopteraCurculionidae

(Marshall, 1948) comb. n.

Calyptopygus ellipticus Marshall, 1948: 466. Syntypes 8, Kambaiti [Kan Paik Ti], Myanmar (BMNH, NHRS).

#### Diagnosis.

*Limnobaris elliptica* is very similar to *Limnobaris babai* from Japan. These two species can be distinguished from all others by the short, curved rostrum, the blunt ventromedian process on the male protibia and almost glabrous elytra. *Limnobaris elliptica* is on average somewhat smaller than *Limnobaris babai* (3.2–3.8 mm vs. 3.5–4.5 mm), has an apically pointed penis (rounded in *Limnobaris babai*) and at least the males have longer setae on the underside of the profemur. *Limnobaris basalis*, another similar species, has a longer rostrum, larger scales on the elytral base and occurs in Fujian. The Nepalese *Limnobaris martensi* is slenderer and almost entirely glabrous.

#### Distribution.

The species is known from three sites west of the Gaoligong Mountains in the border region between Myanmar and Yunnan Province, China (Fig. 13).

#### Biology.

We found larvae, pupae and freshly eclosed adults inside flowering culms of *Scirpus wichurai* Böckeler in late September. A few specimens already had exited the plant and one was observed on a tall *Scleria* species, which does not appear to be a larval host. Many larvae were killed by three to five specimens of an apparently undescribed *Entedon* Dalman species (Eulophidae, Hymenoptera), a large and widespread genus known to parasitize beetle larvae (C. Hansson, *in litt*.). The weevil overwinters and the first specimens appear on the host plant in late March.

#### Material examined.

CHINA. Yunnan: 盈江伐木場 [Famuchang (logging headquarter), Yingjiang ], 1700 m, 13.iv.1980 (IZCAS 3); Xiaozahe, Tengchong County, 1800 m, 26.ix.2013 (IZCAS 4, JPPC 4, ZIN 2). MYANMAR. Kachin: Kambaiti [Kan Paik Ti], Myitkyina District, 2130 m, 28.iii.–3.iv.1934 (3x), 14.iv.1934 (BMNH 4).

### Limnobaris japonica

Taxon classificationAnimaliaColeopteraCurculionidae

Yoshihara & Morimoto, 1994

Limnobaris japonica Yoshihara & Morimoto, 1994: 447. Holotype male, Yunomata, Aomori Pref., Japan (ELKU).

#### Diagnosis.

*Limnobaris japonica* is the only species in Hokkaido, Honshu and the Kuril Islands that has dense lateroventral vestiture. Unlike *Limnobaris dolorosa* and *Limnobaris t-album*, the two other species with this trait, *Limnobaris japonica* has squamiform vestiture also on the prosternum. The penis is similarly elongate as in *Limnobaris t-album* but apically more gradually narrowed.

#### Distribution.

The species occurs in Hokkaido and Honshu (Yoshihara and Morimoto 1994) and Sakhalin Oblast (Egorov et al. 1996) (Fig. 13).

#### Biology.

Adult weevils have been collected from *Carex thunbergii* Steud. and unidentified *Carex* species (Yoshihara and Morimoto 1994).

#### Material examined.

JAPAN. Hokkaido, Fukushima-cho, Sengen, 13.vi.1998 (JPPC 2). RUSSIA. Sakhalin Oblast: Kunashir Island, Yushno-Kurilsk, 15.vi.1991 (ZIN 1).

### Limnobaris kabakovi

Taxon classificationAnimaliaColeopteraCurculionidae

Korotyaev, 1982

Limnobaris kabakovi Korotyaev, 1982: 140. Holotype male, Tam Dao, Tinh Vinh Phuc, Vietnam (ZIN).

#### Diagnosis.

*Limnobaris kabakovi* is a relatively large (4.1–4.6 mm) species with ventral projection on the male protibia and scattered, appressed, squamiform setae on the elytron. Similarly appressed elytral vestiture occurs also in *Limnobaris albosparsa*, but that species is smaller and occurs only around the Eastern Sea. At least the type series of *Limnobaris kabakovi* has brownish elytra.

#### Distribution.

The species is known only from the type locality, a mountain resort and national park in Northern Vietnam (Fig. 13). This is the most southern record for a species of *Limnobaris*.

#### Biology.

Unknown.

#### Material examined.

VIETNAM. Tinh Vinh Phuc: Tam Dao, 25.ii.1962 (ZIN 5).

### Limnobaris kumei

Taxon classificationAnimaliaColeopteraCurculionidae

(Yoshihara & Morimoto, 1997) comb. n.

Calyptopygus kumei Yoshihara & Morimoto, 1997: 1. Holotype male, Mt. Takôyama, Yomitan-son, Okinawa Island, Japan (ELKU).

#### Diagnosis.

*Limnobaris kumei* is the only known species that has neither dense lateroventral vestiture nor a male protibial projection. Moreover, the rostrum is longer and sexually more dimorphic than in other congeners. Females have the antenna inserted in the basal half of the rostrum, a condition otherwise noticed only in *Limnobaris albosparsa* and *Limnobaris tibialis*.

#### Distribution.

The species is known from the Ryūkyū Islands (Okinawa) and Taiwan (Fig. 13).

#### Biology.

Adult weevils were collected from unidentified Cyperaceae (Yoshihara and Morimoto 1997).

#### Material examined.

TAIWAN. [data not recorded] (BPBM 1).

### Limnobaris martensi

Taxon classificationAnimaliaColeopteraCurculionidae

Korotyaev sp. n.

http://zoobank.org/A7C0F62E-6BD3-453B-BB1C-97342A7EBDCC

#### Diagnosis.

**Figure 2.**
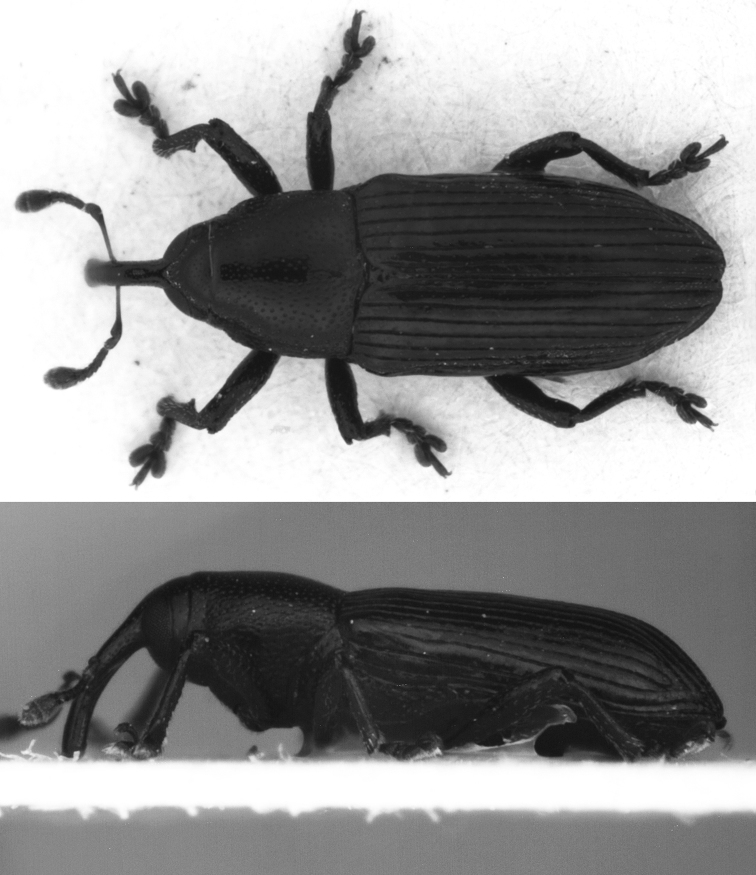
*Limnobaris martensi*, dorsal and lateral view of holotype (L = 3.2 mm).

*Limnobaris martensi* is a shiny, almost entirely glabrous species that has just a few inconspicuous setae on the ventral side and the legs (Fig. 2). The slightly bulging apical section of the tenth interstria is nearly smooth in *Limnobaris martensi* but more or less serrated in the other glabrous species *Limnobaris babai* and *Limnobaris elliptica*. Very slender, abraded *Limnobaris tibialis* can be distinguished from *Limnobaris martensi* by the sharply pointed, more basally inserted male protibial projection and apically round penis (Figs 8, 10).

#### Description.

Rostrum as long as pronotum, weakly and evenly curved, cylindrical, parallel-sided in basal 2/3, slightly dilated to apex; depression before eyes very weak but distinct. Dorsal surface of rostrum evenly convex, with short striole at level of antennal insertion; shiny, with sparse minute round punctures. Sides of rostrum at base with denser and larger elongate punctures. Antennae inserted at 0.57 × length of rostrum from base, antennal scrobe shortly continued beyond base of antenna. Ventral margin of antennal scrobe merging with lateroventral edge of rostrum at half way to eye; dorsal margin of scrobe reaching eye. Scape of antenna slender, shortly widened at apex. First segment of funicle 1.5 × as long as wide, 2nd slightly longer than wide, 3rd weakly transverse, 4–7th moderately to strongly transverse. Base of club very broadly rounded, almost truncate, but clearly separated from broad 7th funicular segment, apex of club broadly rounded. Frons weakly convex, at anterior margin as broad as base of rostrum, slightly widened posteriad, shiny, with sparse small punctures. Vertex with reticulate microsculpture. Eyes large.

Pronotum 1.1 × as wide as long, parallel-sided in basal half, then weakly narrowed to shallow apical constriction. Base of pronotum feebly bisinuate. Disc weekly and evenly convex, sub-matt due to reticulate microsculpture, with rather sparse fine, somewhat angular, round or oblong punctures, separated usually by not less than own diameter, in some places by 2–3 × diameter. Median line without microreticulation and punctures. Scutellum shiny, nearly rectangular, feebly widened at base.

Elytra 1.8 × as long as wide, with well-pronounced humeri, parallel-sided in basal half, rather narrowly rounded at apex; sutural angle slightly sinuate. Disc flattened, with fairly abrupt declivity; preapical prominences very distinct. Striae deep and narrow, intervals flat, about 4 × as wide as striae, shiny, with 1 row of small punctures and much finer microreticulation than on pronotum. Intervals in many places distinctly impressed around sparse and inconspicuous punctures in striae.

Legs slender and fairly long. Fore tibia with large tooth slightly proximal of middle of inner surface, apex of tooth blunt (Fig. 4). 1st tarsite 1.5 × as long as wide, 2nd clearly transverse, rounded at sides, 3rd in fore tarsus 1.7 × as broad as 2nd. 5th tarsite slender, weakly widened toward apex, by one-half of its length projecting beyond lobes of 3rd tarsite. Length of claw 1.5 × width of claw-segment at apex. Penis as in Fig. 8, moderately bent ventrally at base and apex, basal apodemes shorter than in other species.

**Figures 3–5.**
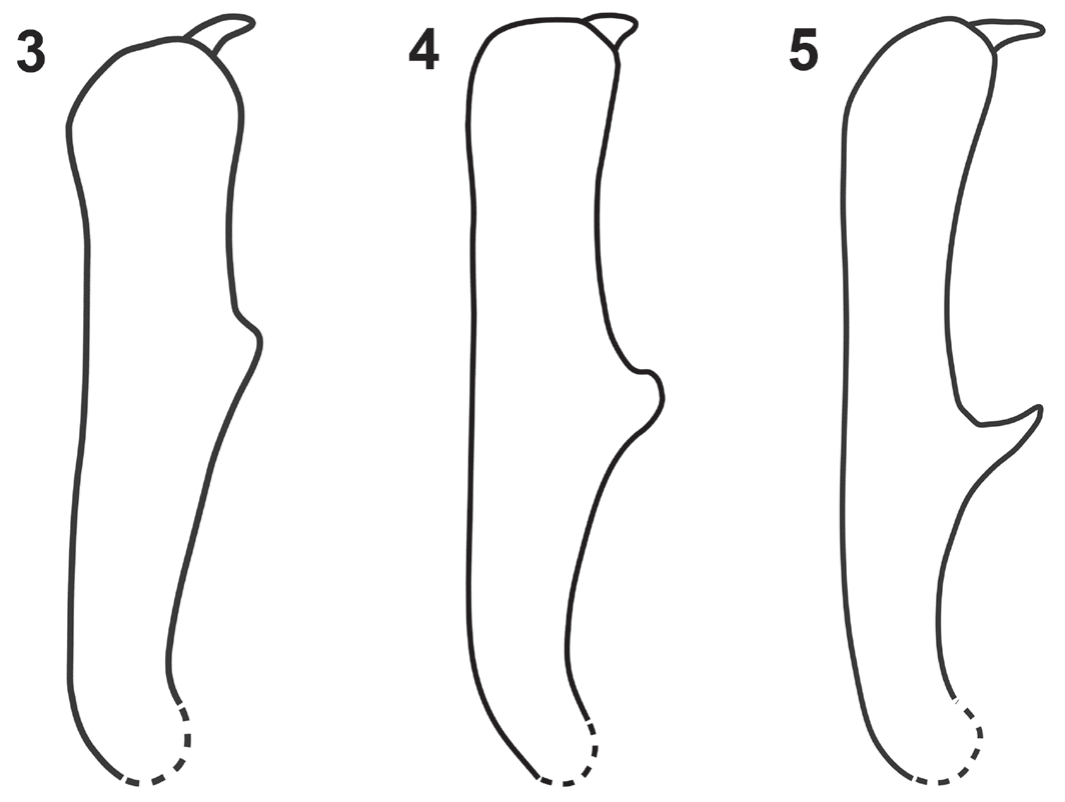
Male protibia of *Limnobaris* species. **3** *Limnobaris basalis* **4** *Limnobaris martensi* **5** *Limnobaris tibialis*.

**Figures 6–12.**
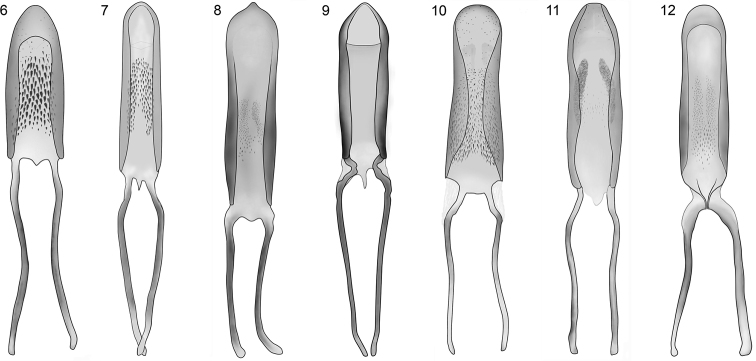
Penis of *Limnobaris* species, dorsal view. **6** *Limnobaris dolorosa* **7** *Limnobaris t-album* **8** *Limnobaris martensi* **9** *Limnobaris elliptica* **10** *Limnobaris tibialis* **11** *Limnobaris basalis* **12** *Limnobaris kabakovi*.

Body black; scape of antenna in basal 2/3 light brown, apical third of scape, 1st and base of 2nd segments of antennal funicle, and tarsi dark brown, humeral callus brownish. Upper side bare, legs with sparse short recumbent white setae, sides of abdomen with few inconspicuous setae.

Length of body 3.2 mm, width at shoulders 1.15 mm.

#### Distribution.

**Figure 13.**
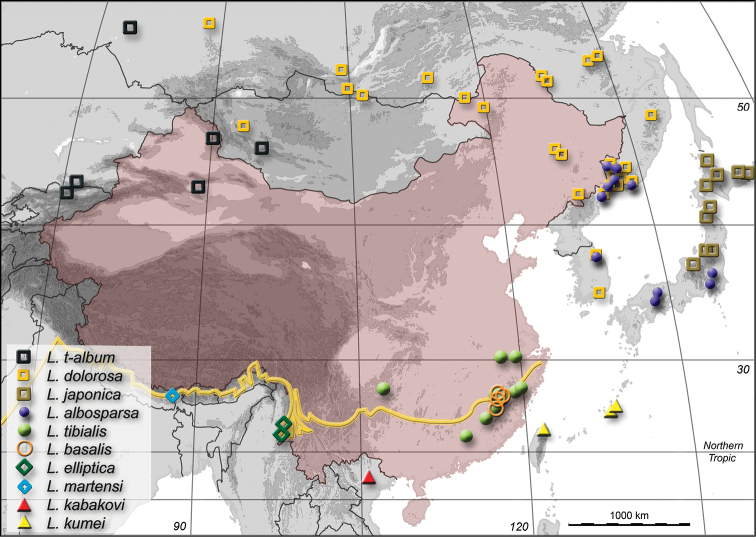
Map of China and neighboring countries showing the distribution of ten of eleven Palaearctic *Limnobaris* species. See Yoshihara and Morimoto (1994) for distribution of *Limnobaris babai* in Japan. Yellow line demarks southern limit of Palaeartic Realm according to Hoffmann (2001). Topographic relief in 1000 m increments calculated from GTOPO30 data set of US Geological Survey, EROS data center.

The only known specimen is from Eastern Nepal (Fig. 13).

#### Biology.

Unknown.

#### Material examined.

Holotype male: Nepal, 272, Taplejung Distr., Kabeli Khola, N Yamputhin, S-Hang, 1700–2200 m, Kulturland/Busch, 5 Sep. 1983, J. Martens and B. Daams (SMNS).

#### Etymology.

The species is named after Dr. Jochen Martens (Mainz).

### Limnobaris tibialis

Taxon classificationAnimaliaColeopteraCurculionidae

(Voss, 1953) comb. n.

Pertorcus tibialis Voss, 1953: 75. Holotype male, Kuatun [Guadun], Fujian, China (AKMB).
Pertorcus tibialis pilifer Voss, 1958: 76. Holotype male, Kuatun [Guadun], Fujian, China (AKMB). syn. n.

#### Diagnosis.

*Limnobaris tibialis* is a small species (2.1–3.1 mm) with sparse ventrolateral vestiture and erect setae on the elytron. Males have a sharply pointed, anteriorly directed process on the ventral edge of the protibia, which is located more basally than the blunt tooth of other species (Fig. 5). *Limnobaris albosparsa*, the only other species with a sharply pointed process, has a slightly wider penis basally, appressed rather than erect setae on the elytron and is allopatric. The number and width of white setae on the elytral interstriae vary considerably even in series taken at the same locality. Females may be confused with sympatrically occurring *Limnobaris basalis*, but the latter species has white setae crowded at the elytral base and is slightly less elongate. Most female *Limnobaris tibialis* can be distinguished by more basally inserted antennae.

#### Notes.

Voss (1958) distinguished a pilose “form” with slightly different protibial process. Even though the size of setae can differ noticeably between individual specimens, transitional forms occur even in the same series. *Pertocus tibialis pilifer* is a new junior synonym of the nominal species.

#### Distribution.

The species is known from Southeast China (Anhui, Fujian, Guizhou, Jiangxi, Zhejiang) (Fig. 13).

#### Biology.

In Northern Fujian, we found this species on several, ca. 20–30 cm tall *Carex* species. The larva develops in the basal, ca. 2 mm wide section of the culm. Pupation starts in July and newly eclosed adults appear in the same month.

#### Material examined.

CHINA. Anhui: Taipingshien, x.1932 (MCZ 30+). Fujian: Guadun, Wuyi Mts., 3.iv.1938 (PT of P. t. pilifer), 5.iv.1938 (HT + 2 PT of P. t. tibialis + 1), 8.iv.1938 (4x), 18.iv.1938 (1 PT of P. t. tibialis + 1 PT of P. t. pilifer), 20.iv.1938 (HT of P. t. pilifer), 5.v.1938 (2x), 26.v.1938 (PT of P. t. pilifer) (AKMB 15); ditto, 3.iii.1938 10.iv.1938 (PT of P. t. tibialis), 10.iv.1938 (PT of P. t. tibialis) (ZIUH 2); ditto, 7.iv.1938, 18.iv.1938 (2x) (CWOB 3); Changting, Hotien, 15.iv.1941 (BPBM 1), Changteh, Talungchan, 15.iv.1941 (BPBM 1); Daanyuan, Wuyi Mts., 24.–27.iv.2013 (BMNH 4, IZCAS 21, JPPC 12, ZIN 4), 16.–19.vii.2013 [larvae, pupae] (JPPC); 将乐龙栖山余家坪 [Yujiaping, Longxi Mts., Jiangle], 11.iv.1991 (IZCAS 2); 将乐龙栖山里山 [Lishan, Longxi Mts., Jiangle], 22. iv.1991 (IZCAS 1); 建阳黄坑桂林 [Guilin, Huangkeng, Jianyang], 11.iv.1960 (IZCAS 1); 建阳 [Jianyang], 10.v.1965 (IZCAS 1); 邵武铁洋 [Tieyang, Shaowu], 9.vi.1965, 28.vi.1965 (IZCAS 2); Shaowu, Wuyi Mts., 30.iv.1943 (BPBM 1). Guizhou: 遵义市绥阳县宽阔水自然保护区香广山村[Xiangguangshan village, Kuan Kuoshui Natural Reserve, Suiyang, Zunyi], 4.vi.2010 (IZCAS 1). Jiangxi: 九连山保护区 [Jiu Lianshan Natural Reserve], 29/30.ix.1979 (IZCAS 2). Zhejiang: 临安市西天目山禅院寺[Chanyuan Temple, West Tianmu Mts., Lin’an], 22.viii.2009 (IZCAS 2); 景宁 [Jingning], 28.vii.2010 (ZAFU 1); 庆元百山祖 [Baishanzu, Qingyuan], 16.v.1994 (IZCAS 2).

### Limnobaris t-album

Taxon classificationAnimaliaColeopteraCurculionidae

(Linnaeus, 1758)

Curculio t-album Linnaeus, 1758: 379. Type male, Westerbothnia [=Västerbotten], Sweden (LSUK).
Curculio nigrinus Herbst, 1795: 60. Lectotype designated by Dieckmann (1991: 307), female, collecting data unknown (MNKB). Synonymized with *Limnobaris t-album* by Gemminger and Harold (1871) and Prena (2008), and with *Limnobaris t-album atriplicis* sensu Dieckmann by Dieckmann (1991).
Curculio hypoleucus Marsham, 1802: 274. Type probably auctioned in 1820 (R. Thompson in Dieckmann 1991); synonymized by Gyllenhal (1813) but identity unknown; synonymy with *Limnobaris dolorosa* by Dieckmann (1991: 310, not 307) unintended but possibly correct.
Baridius crocopelmus Gyllenhal in Schönherr 1836: 720. Holotype, sex not determined, Elisabethgrad [=Kirovograd], Ukraine (NHRS). Synonymized with *Limnobaris t-album* by Gemminger and Harold (1871) but reinstated or overlooked by subsequent authors. Reestablished synonymy.
Baridius pusio Boheman in Schönherr 1844: 173. Holotype female, Sicily, Italy (NHRS). Synonymized with *Limnobaris t-album* by Gemminger and Harold (1871) but reinstated or overlooked by subsequent authors; synonymized with *Limnobaris t-album atriplicis* sensu Dieckmann by Dieckmann (1991) and with *Limnobaris t-album* by Prena (2008).
Baris t-album sculpturata Faust, 1885: 201. Lectotype designated by Dieckmann (1991: 309), male, Ysyk-Köl, Kyrgyz Republic (SNSD). Erroneously synonymized with *Limnobaris dolorosa* by Dieckmann (1991). syn. n.
Limnobaris bedeli Reitter, 1888: 274. Holotype female, Lenkoran, Azerbaijan (SNSD). syn. n.
Limnobaris scutellaris Reitter, 1888: 273. Type not located [not in HNHM], Utsch-Dere, Krasnodarsky Kraj, Russia. syn. n.
Baridius (Limnobaris) martulus J. Sahlberg, 1892: 223. Lectotype designated by Dieckmann (1991: 309), sex not determined, Jakobstad, Finland (MZH). Synonymized with *Limnobaris t-album* by Champion (1905).
Limnobaris sahlbergi Reitter, 1901: 82. Syntypes at least 4, Ysyk-Köl, Tschüi River, Kyrgyz Republic (BMNH, HNHM, SDEI). syn. n.
Limnobaris reitteri Munster, 1928: 281. Replacement name for *Limnobaris pusio* sensu Reitter (1895), not Boheman (1844). Synonymized with *Limnobaris t-album atriplicis* sensu Dieckmann by Dieckmann (1991) and with *Limnobaris t-album* by Prena (2008).
Limnobaris t-album atriplicis sensu Dieckmann (1991): misinterpretation of *Curculio atriplicis* Fabricius, 1777 (= *Curculio laticollis* Marsham, 1802; nomen protectum) introduced into the literature by invalid lectotype designation (see Prena 2008).

#### Diagnosis.

Diagnostic characters for the distinction of *Limnobaris t-album* and two other species with dense lateroventral vestiture are given under *Limnobaris dolorosa*.

#### Notes.

The squamiform setae on the first two ventrites and the metasternum vary in size and density between local populations. Specimens with nearly imbricate setae have been recognized by some authors either as a subspecies or species. Dieckmann (1991) considered them as a subspecies because he recongized (1) a continuous northern distribution of the nominal form of *Limnobaris t-album* and (2) a relatively narrow transitional zone to the deviating southern form. Prena (2008) rejected the subspecies because the applied name was based on an invalid lectotype designation and the transitional zone corresponds closely with the winter isotherm indicating a possible environmental effect on the scale pattern. The issue needs to be readdressed with appropriate methods in a larger context, *i.e.* under inclusion of the Central Asian population named *Limnobaris sahlbergi* and *Limnobaris sculpturata*. Until this is accomplished, we suggest to include all nominal taxa with a t-album-type of aedeagus under *Limnobaris t-album*. The type repository of *Limnobaris scutellaris* Reitter, described from Utsch-Dere in South Russia, remains unknown. The herein proposed synonymy with *Limnobaris t-album* is based on Reitter’s comparison with that species.

#### Distribution.

*Limnobaris t-album* is widespread in the Palaearctic Region and apparently extends further north than the other common species, *Limnobaris dolorosa*. In fact, *Limnobaris t-album* is the only baridine weevil worldwide that reaches either of the polar circles. The most eastern records are from Mongolia (ca. 95°E), Xinjiang Province, China (ca. 90°E) and the adjacent Russian territory (Legalov 2010).

#### Biology.

Because of taxonomic confusion with *Limnobaris dolorosa*, published life history data are unreliable and need verification. The adult occurs on *Carex acutiformis* Ehrh. between middle May and early July in Northern Germany (J. Prena, unpubl. data). *Carex atherodes* Spreng., *Carex pseudocyperus* L., *Carex rostrata* and *Scirpus sylvaticus* grew occasionally nearby but received no attention by the weevil. Reports from *Carex rostrata*, *Carex vesicaria* L. and the tall sedges *Cladium mariscus* and *Schoenoplectus lacustris* (*i.e.*, Heyden in Brisout 1870, Heyden 1877, Sahlberg 1892, Kleine 1910, Hoffmann 1955, Cawthra 1957, Scherf 1964) may apply to *Limnobaris dolorosa*. Associations with plants other than sedges are accidental.

#### Material examined.

CHINA. Xinjiang: 青河县达巴特新村 [Dabate, Qinghe], 7.vii.2009 (IZCAS 1); Turpan (HNHM 1). KAZAKHSTAN. Aulie-Ata [=Taraz] (HNHM 3); Wernyi [=Almaty] (HNHM 1, SFFM 6). KYRGYZSTAN. Bishkek (HNHM 2); Ketmen’tebe (HNHM 1); Tschu [Tschüi] River, Issyk-kul [Ysyk-Köl] (SDEI 1); Yssik-kul [Ysyk-Köl] (SNSD 3). MONGOLIA. Sargyn-Gobi, S Som. Sarga, Aimak Gobi Altai, 970 m, 18.–20.vi.1964 (MNKB 1). RUSSIA. Novosibirskaya Oblast: Kuibyshev Distr., Zonovo, 29.v.1961 (IZCAS 1). UZBEKISTAN. Jizzakh Prov.: Djizak [Jizzakh] (SNSD 1).

### Key to species

**Table:** 

1	Lateral parts of mesothorax, metathorax and abdomen densely covered with whitish or yellowish squamiform setae (Fig. 1); male protibia without ventromedian projection	2
–	Lateral parts of mesothorax, metathorax and first three ventrites with widely spaced slender to moderately wide setae, derm partially uncovered, remaining underside with sparse hairlike setae; male protibia with ventromedian projection except in *Limnobaris kumei*	4
2	Mesoscutellum with lateral portions squamose; prosternum with yellowish white, squamiform setae similar to those on meso- and metasternum; elytron with squamiform setae restricted to basal third of interstriae 3–6; Japan, Russia (Kuril Islands)	*Limnobaris japonica*
–	Mesoscutellum glabrous; prosternum with dingy white, slender setae dissimilar to those on meso- and metasternum; elytron with squamiform setae more evenly distributed if present; Palaearctic Region except Pacific offshore islands	3
3	Mesosternum, metasternum and abdominal ventrites laterally with wide, imbricate setae completely covering integument; male with ventrites 1–2 strongly depressed; penis shorter and wider (Fig. 6); transpalaearctic	*Limnobaris dolorosa*
–	Mesepisternum, mesepimeron and metepisternum with imbricate scales forming T-shaped pattern, adjacent sclerites (flanks of metasternum, often also first two ventrites) less densely covered with wide setae, leaving integument in between visible; male with ventrites 1–2 slightly depressed; penis longer and narrower (Fig. 7); Western and Central Palaearctic Region	*Limnobaris t-album*
4	Elytron with squamiform setae only at base or entirely glabrous	5
–	Elytron with squamiform setae scattered throughout (setae occasionally sparse or absent in abraded *Limnobaris tibialis*)	8
5	Squamiform setae at elytral base conspicuous, *ca.* as long as interstrial width; male ventrotibial process distally of mid-length of tibia (Fig. 3); total length 2.3–3.1 mm; East China (Fujian)	*Limnobaris basalis*
–	Squamiform setae at elytral base inconspicuous or entirely absent; male ventrotibial process at mid-length of tibia (Fig. 4); total length >3.1 mm; other distribution	6
6	Body very slender, elytra *ca.* 2.0 × as long as wide; apical margin of elytron smooth in dorsal view; Nepal	*Limnobaris martensi*
–	Body stouter, elytra <1.8 × as long as wide; apical margin of elytron serrate in dorsal view; more eastern distribution	7
7	Apical margin of elytron distinctly serrate in dorsal view; profemur of fresh specimens ventrally with curved setae longer than tarsal claw; body length 3.2–3.8 mm; penis triangularly narrowed and pointed apically; China (Yunnan), Myanmar	*Limnobaris elliptica*
–	Apical margin of elytron indistinctly serrate in dorsal view; profemur of fresh specimens ventrally with curved setae shorter than tarsal claw; penis rounded apically; body length 3.5–4.5 mm; Japan	*Limnobaris babai*
8	Rostrum long (male *ca.* 1.3 ×, female *ca.* 1.5 × as long as pronotum); funicular joint 7 elongate; male protibia without ventromedian projection; Ryūkyū Islands and Taiwan	*Limnobaris kumei*
–	Rostrum moderate (male <1.1 ×, female <1.3 × as long as pronotum); funicular joint 7 transverse; male protibia with ventromedian projection; other distribution	9
9	Elytron with erect squamiform setae; central East China (Anhui, Fujian, Guizhou, Jianxi, Zhejiang)	*Limnobaris tibialis*
–	Elytron with appressed squamiform setae; northern or southern East Asia	10
10	Body length <3.5 mm; rostrum at least as long as pronotum; southern continental Russian Far East, Korean Peninsula and Japan	*Limnobaris albosparsa*
–	Body length >4.0 mm; rostrum shorter than pronotum; Vietnam	*Limnobaris kabakovi*

## Supplementary Material

XML Treatment for Limnobaris albosparsa

XML Treatment for Limnobaris babai

XML Treatment for Limnobaris basalis

XML Treatment for Limnobaris dolorosa

XML Treatment for Limnobaris elliptica

XML Treatment for Limnobaris japonica

XML Treatment for Limnobaris kabakovi

XML Treatment for Limnobaris kumei

XML Treatment for Limnobaris martensi

XML Treatment for Limnobaris tibialis

XML Treatment for Limnobaris t-album
